# Cytomegalovirus and Epstein–Barr Virus Associations with Neurological Diseases and the Need for Vaccine Development

**DOI:** 10.3390/vaccines8010035

**Published:** 2020-01-20

**Authors:** Peter A. C. Maple

**Affiliations:** Clinical Neurology Research Group, Division of Clinical Neuroscience, School of Medicine, Queen’s Medical Centre, University of Nottingham, Nottingham NG7 2UH, UK; eastyorksmicrobiol@gmail.com; Tel.: +44-115-8231443; Fax: +44-115-9709738

**Keywords:** Epstein–Barr virus, Cytomegalovirus, multiple sclerosis, congenital infection

## Abstract

Herpesviruses have been isolated from a wide range of hosts including humans—for which, nine species have been designated. The human herpesviruses are highly host adapted and possess the capacity for latency, allowing them to survive in the host for life, effectively hidden from the immune system. This ability of human herpesviruses to modulate the host immune response poses particular challenges for vaccine development but at the same time proves attractive for the application of human herpesvirus vaccines to certain spheres of medicine. In this review, congenital cytomegalovirus (CMV) infection and hearing loss will be described followed by a comment on the status of current vaccine development. Secondly, the association of Epstein–Barr virus (EBV) infection with multiple sclerosis (MS) and how EBV vaccination may be of benefit will then be discussed. Prevention of congenital CMV by vaccination is an attractive proposition and several vaccines have been evaluated for potential use. Particularly challenging for the development of CMV vaccines are the needs to prevent primary infection, reinfection, and reactivation at the same time as overcoming the capacity of the virus to generate highly sophisticated immunomodulatory mechanisms. Cost and the practicalities of administering potential vaccines are also significant issues, particularly for low- and middle-income countries, where the burden of disease is greatest. An effective EBV vaccine that could prevent the 200,000 new EBV-associated malignancies which occur globally each year is not currently available. There is increasing interest in developing EBV vaccines to prevent MS and, in view of the association of infectious mononucleosis with MS, reducing childhood infectious mononucleosis is a potential intervention. Currently, there is no licensed EBV vaccine and, in order to progress the development of EBV vaccines for preventing MS, a greater understanding of the association of EBV with MS is required.

## 1. Introduction

Cytomegalovirus (CMV) and Epstein–Barr virus (EBV) are human herpesviruses belonging to the family *Herpesviridae*, which, as of 2018 [1], comprises 122 species grouped into 19 genera, three subfamilies and three families. Herpesviruses have been isolated from a wide range of hosts (e.g., mice, fish, birds, elephants, macaques, and bovines) including humans—for which nine species have been designated. The human herpesviruses are highly host adapted [2] and possess the capacity for latency [3], allowing them to survive in the host for life, effectively hidden from the immune system. This ability of human herpesviruses to modulate the host immune response [4] poses particular challenges for vaccine development [5] but at the same time proves attractive for the application of human herpesvirus vaccines to certain spheres of medicine [6,7].

The human herpesviruses are responsible for a wide range of pathologies and, currently, effective vaccination is available for only one of them, the varicella-zoster virus [8]. In this short review, the roles of CMV and EBV will be described in two diseases of neurological interest. Firstly, congenital CMV infection and hearing loss will be described followed by a comment on the status of current vaccine development. Secondly, the association of EBV infection with multiple sclerosis and how EBV vaccination may be of benefit will then be discussed.

## 2. Congenital Cytomegalovirus Infection and Hearing Loss

Cytomegalovirus (CMV) infection is usually acquired during the early years of life and, in immunocompetent individuals, it is usually subclinical. In immunocompetent adults, CMV infection is most commonly associated with abnormal liver function test results, malaise and fever [9] and it is the second leading cause of infectious mononucleosis behind EBV [10]. CMV infection or reactivation is responsible for significant morbidity and mortality in the immunocompromised [11], solid organ transplant recipients [12] and critically ill immunocompetent patients [13]. Finally, congenital CMV infection can have devastating consequences for the neonate including growth and development abnormalities such as microcephaly, hepatosplenomegaly, chorioretinitis, and sensorineural hearing loss [14]. In many countries, CMV screening is not undertaken during pregnancy as such an activity is viewed as of dubious clinical benefit considering the lack of vaccines and acceptable treatment options available [15]; however, this perspective is being challenged [16]. Worldwide, CMV congenital infection is highly variable with estimated rates of 0.6 to 0.7% of live births in industrialized countries and 1 to 5% in developing countries [17].

Congenital CMV infection can follow infection during pregnancy of a previously uninfected gravida (primary infection), or be due to reactivation of CMV from the latent state with in utero transmission, or may arise following reinfection with a different CMV strain. The virus is shed in body fluids (e.g., saliva, urine, breast milk, and tears) and close personal contact is the main vehicle of transmission in most individuals; however, transplacental infection of the foetus, infection at birth through contact with the virus in the genital tract, and transfusion-acquired infection from infected blood can also occur [18]. In primary infection, CMV is seeded throughout the body following infection of epithelial cells [19] and myeloid-lineage leukocytes [20]. Innate immune responses (e.g., natural killer cells) followed by adaptive humoral and cell-mediated immune responses combine in the healthy host to limit virus multiplication. In particular, functional CD8^+^/CD4^+^ T cell responses are critical for controlling virus replication [21]; however, the virus is not fully cleared, and latency is established in cells (e.g., CD34 progenitors) of the myeloid lineage [22]. CMV infection or reactivation in disrupted or immature immune systems can result in uncontrolled virus multiplication with attendant morbidity and mortality: haematopoietic stem cell transplant represents the former [23], and congenital infection represents the latter.

The prevalence of CMV infection, as demonstrated by seroprevalence studies, is linked with geography, ethnicity and race. At a macro-geographical level, many studies in African countries report CMV seroprevalences in excess of 80% even in children [24], while significantly lower CMV seroprevalences of 59% and 15% have been reported in the UK for adults and young children, respectively [25,26]. Within national boundaries CMV seroprevalences can vary widely; for example, in Bradford, a city in northern England, CMV seroprevalence was 49% among pregnant white women and 89% among South Asian women [27]. The findings of seroprevalence studies have also been replicated in studies of congenital CMV prevalence [28], which have also shown associations with geographical location [29], ethnicity [30] and race [31].

Congenital hearing loss is one of the most prevalent chronic conditions affecting children and, in several countries, universal screening during the first month following birth have been introduced [32]. Apart from genetic causes, congenital CMV is now the most common cause of sensorineural hearing loss in newborns in countries which have eliminated rubella [33]. Primary maternal infection carries the highest risk of symptomatic disease at birth and the earlier in pregnancy infection occurs the greater the risk of adverse sequelae [34]. Rates of congenital CMV have been estimated at between 0.4% and 6.0% and vary depending upon maternal CMV seroprevalence and study design [35]. Most newborns infected with CMV are asymptomatic and develop normally; however, approximately 10–15% present with clinically apparent/symptomatic disease including hearing loss [36]. A small number of asymptomatic and symptomatic infants will subsequently develop late onset hearing loss. Goderis and colleagues [37] have reported delayed-onset hearing loss in 10.6% of children born with symptomatic congenital CMV and 7.8% in congenital CMV children asymptomatic at birth. Although the likelihood of hearing loss is less in newborns who are asymptomatic at birth, the much higher prevalence of asymptomatics versus symptomatics translates to children with no evidence of hearing loss during the perinatal period ultimately being a significant reservoir of cases of hearing loss. Another factor influencing the population burden of hearing loss is maternal CMV seroprevalence. Although there is a much greater risk of congenital CMV following primary infection during pregnancy, it has been estimated that only a small number (1%–4% depending upon population group) of non-immune gravidas contract primary infection during this time [38]. Non-primary maternal infection following CMV reactivation or reinfection has been shown [39] to be responsible for 77.3% cases of congenital CMV.

The diagnosis and prevention of congenital CMV presents several challenges. Firstly, maternal CMV infection is difficult to diagnose due to the lack of signs and symptoms in most cases, and in those cases where they do occur, they are non-specific. Secondly, approximately 50% of gravidas will have already been infected and there is a need to differentiate recently produced specific antibody from that associated with previous infection. CMV specific immunoglobulin M (IgM) detection can be useful in supporting a diagnosis of recent infection, but assays tend to lack sensitivity and specificity [40]; furthermore, false positivity of CMV IgM assays in detecting primary infection is a significant problem [41]. Measurement of CMV specific immunoglobulin G (IgG) avidity [42] can be used to differentiate newly formed low avidity antibody consistent with recent infection and combining CMV IgM and avidity detection using validated assays for the diagnosis of primary infection is recommended [43]. Unfortunately, as many cases of congenital CMV-mediated hearing loss follow non-primary infection the detection of specific IgG and IgM markers has limited clinical utility. In those patients where there is clinical suspicion of CMV infection the detection of specific antibody markers consistent with recent infection may aid with differential diagnosis and present evidence of a higher risk for symptomatic congenital infection [44].

Foetal CMV infection can be confirmed with high positive predictive values [45,46] by isolation of virus or detection of viral DNA from amniotic fluid. False-negative results may occur up to 20 weeks’ gestation because the virus may not be excreted in the urine of the foetus [47] or if sampling is less than six weeks following the initial maternal infection [48]. Detection of congenital CMV infection in the neonate needs to be undertaken within three weeks of birth due to the possibility of postnatal infection; for instance, from breast milk [49], and to facilitate prompt interventions [50]. The most suitable diagnostic methodology is real-time polymerase chain reaction using saliva specimens [51,52,53]. Saliva [54] or dried blood spot PCRs [55] have been used in support of newborn hearing loss screening programmes as they offer the capacity to target asymptomatic neonates “at risk” of developing hearing loss and who may benefit from antiviral treatment or other intervention measures [56,57,58].

Antiviral treatment of symptomatic congenital CMV central nervous system (CNS) disease is recommended; however, treatment options are very limited and constrained by drug toxicity, cost, and the need for evidence-based efficacy data [59]. Initially, Ganciclovir treatment (6 mg/kg per dose IV every 12 h for 6 weeks) was shown by a Collaborative Antiviral Study Group (CASG) of the National Institute of Allergy and Infectious Diseases (NIAID) to prevent hearing deterioration based on an improvement of brainstem-evoked response audiometry of one gradation for cases presenting with hearing loss or no change from baseline in cases presenting with normal hearing [60]. In the CASG study [60], CMV viral loads were not measured and neutropenia was evident in approximately two-thirds of treated infants. Subsequently, the Ganciclovir oral pro-drug Valganciclovir has been shown to be an acceptable alternative [61]. Recently, treatment with Valganciclovir for six months has been shown by a further NIAID CASG study [62] to produce some additional improvement in longer-term hearing and development outcomes compared with the six-week course of therapy. Several retrospective case studies [63,64,65] have presented additional evidence supporting the potential benefits of prolonged courses of Valganciclovir treatment leading to the current acceptance that six month’s treatment is optimal subject to an evaluation of risks versus benefits [66,67,68,69].

The immune response to CMV has been shown to be broad spectrum and complex, with both innate and adaptive components playing major roles and the virus having evolved several mechanisms in an attempt to circumvent or modulate them [70]. In the immunocompetent host, effective control of CMV is achieved as evidenced by the fact that most cases are asymptomatic. Key host immunological components that contribute towards the effective control of CMV infection include the release of proinflammatory cytokines and chemokines via the toll-like receptor2 (TLR2) pathway following the recognition of virus surface glycoproteins [71], the production of type 1 interferon and activation of natural killer cells [72], the generation of CMV specific CD4^+^/CD8^+^ T cell responses and the production of neutralizing antibodies [73,74]. 

Prevention of congenital CMV by vaccination is an attractive proposition and several vaccines have been evaluated for potential use in this setting (Table 1).

Particularly challenging for the development of CMV vaccines are the needs to prevent primary infection, reinfection, and reactivation at the same time as overcoming the capacity of the virus to generate highly sophisticated immunomodulatory mechanisms [81]. Historically, the first CMV vaccines were developed during the 1970s and were based on attenuated virus strains AD-169 and Towne; however, over time a multiplicity of vaccine candidates have been developed [82]. Several live virus vaccines have been evaluated, comprehensively reviewed by Gerna and Lilleri [83], in which various approaches (e.g., genetically modified V160 vaccine, Towne/Toledo recombinant chimera vaccines, viral vectored vaccines, and Alphavirus replicon particles vaccines) have been used to achieve satisfactory immunogenicity and safety profiles. Similarly, several non-living CMV vaccine candidates have been developed and evaluated [84] including recombinant subunit [85], DNA [86], virus-like particle [87], and peptide [88] vaccines. Currently, there is no licensed CMV vaccine and the furthest progressed vaccine candidate is the recombinant glycoprotein B vaccine which has been assessed in three independent phase II trials [89]. The cost [90] and the practicalities of administering potential CMV vaccines are also significant issues, particularly for low- and middle-income countries, where the burden of disease is greatest [91].

## 3. The Association of Epstein–Barr Virus Infection and Multiple Sclerosis

Epstein–Barr virus (EBV) is a γ-herpesvirus possessing oncogenic activity. It was first described by Epstein, Achong and Barr in 1964 [92] following the isolation of virus particles from lymphoblasts cultured from a patient with Burkitt’s lymphoma. We now know that EBV transforms B cells and is associated with several different lymphoid and epithelial malignancies [93]. Currently, EBV causes approximately 1.5% of cancers worldwide and there has been considerable interest in elucidating EBV oncogenic processes and developing vaccines to prevent EBV mediated disease which has been reviewed elsewhere [94].

In multiple sclerosis, another form of interaction of EBV infection with the host has been observed [95]. Multiple sclerosis (MS) is a chronic, inflammatory, demyelinating, neurodegenerative disease which has an autoimmune component [96]. Most cases of MS initially present with periods of relapse followed by partial healing; however, this relapsing and remitting form of disease (RRMS) gradually becomes increasingly progressive due to accumulating nerve damage and the patient transitions to secondary progressive MS [97,98]. In a small number of cases, approximately 10%–15%, there is no RRMS stage and the patient has ongoing progressive disease—primary progressive MS [99]. Over 120,000 people in the UK live with MS [100] and worldwide it is estimated that approximately two million live with the disease [101]. Effective treatments are available for RRMS [102] but are mostly lacking for progressive disease [103].

Several environmental [104] and genetic factors [105] are known to contribute to an increased likelihood of developing MS. There is extensive evidence that both exposure to EBV [106,107] and a history of infectious mononucleosis [108] are associated with the development of MS. Despite the evidence of an association of EBV infection with MS, the mechanism by which this is achieved remains to be determined. People with MS (PwMS) display a different immune response to EBV infection compared to healthy controls; for example, EBNA-1 IgG levels are higher both in adults and children with MS [109,110]; furthermore, it has been suggested that the humoral response to EBV in MS is associated with disease activity [111]. There is increasing evidence that virus driven immunopathological processes may contribute to MS [112] and it has been shown [113] that EBV-specific CD8 T cells selectively infiltrate the brain in MS and interact locally with virus infected cells.

In view of the possible involvement of EBV infection as a risk factor for MS, and a driver of the MS disease process, EBV specific immunotherapy, EBV antiviral therapy, and EBV immunization are potential intervention measures. A small clinical trial [114] of 13 people with MS who received EBV specific T cell therapy documented both symptomatic and objective neurological improvement in six individuals and the results of further studies are awaited with interest. Few anti-herpetic agents have acceptable activity and toxicity profiles for the treatment of EBV infections [115] and no specific antivirals are recommended for the treatment of EBV infections in people with MS; however, in an anecdotal report [116] clinical improvement in an individual with MS followed treatment with an anti-retroviral agent possessing anti-EBV activity. An effective EBV vaccine that could prevent the 200,000 new EBV-associated malignancies which occur globally each year is not currently available despite the considerable efforts expended in developing EBV gp350 vaccines [117]. There is increasing interest in developing EBV vaccines to prevent MS and, in view of the association of infectious mononucleosis with MS, reducing childhood infectious mononucleosis is a potential intervention [118]. Several EBV vaccines have been evaluated for preventing EBV infection and protecting against infectious mononucleosis [119]. The first vaccine trial in humans was undertaken by Gu and colleagues [120] using a live recombinant vaccinia virus/major EBV membrane antigen BNLF-1 MA (gp 220–340) construct. The authors claimed to show that for the first time it was possible to protect against and/or delay EBV infection by the natural route. To date, the most advanced study of the safety, immunogenicity and efficacy of an EBV vaccine has been reported by Sokal and colleagues [121]. In this study (NCT00430534), undertaken between October 2001 and December 2003, a group of 181 EBV seronegative volunteers between 16 and 25 years of age received three doses of a recombinant gp350 vaccine or placebo. The authors claimed the vaccine to have demonstrable efficacy (mean efficacy rate, 78.0% [95% confidence interval: 1.0–96%]) and that there were no concerns regarding safety or immunogenicity. Alternative EBV vaccines have been evaluated in Phase 1 clinical trials; for example, a virus-like particle EBV vaccine [122] and a CD8^+^ T-cell peptide epitope-based vaccine [123]. Currently, there is no licensed EBV vaccine and, in order to progress the development of EBV vaccines for preventing MS, a greater understanding of the association of EBV with MS is required [124].

## 4. Final Comments

Both cytomegalovirus and Epstein–Barr virus by virtue of their complex interactions with the human host pose several challenges towards the development of safe and effective vaccines. Firstly, their contributions to disease processes remain to be fully determined. In the case of congenital CMV, disease may not only follow primary infection but also be a consequence of reactivation or reinfection and the mechanisms responsible together with protective correlates require further examination [125]. Even in the case of the established CMV glycoprotein B vaccine, protection is incomplete [76] and may not be dependent on gB neutralizing antibodies [126]. At a wider level, CMV may play a role in other diseases such as glioblastoma [127] and arteriosclerosis [128] and the potential benefits of CMV vaccines remain to be determined. The absence of Phase 3 clinical trial data of CMV vaccines is proving a significant limitation to progress. Similarly, the mechanism by which EBV infection, particularly in older children, contributes to multiple sclerosis remains to be described. Frankly, the results of EBV vaccine studies in humans to present have been disappointing. Again, at a wider level, EBV infection may contribute to several autoimmune disease processes [129] and the potential benefits of vaccination are in need of further investigation. Finally, both CMV and EBV infections are widespread in human populations and they contribute a significant burden of disease. Continuing efforts to further understand their contributions to disease processes and the development of safe and effective vaccines are highly justified.

## Figures and Tables

**Table 1:** Reported clinical trials of several vaccine candidates with potential for the prevention of congenital cytomegalovirus (CMV).

CMV Vaccine Candidate	Description of Study (Reference)	Outcome(s)
Conditionally replication defective human CMV vaccine expressing CMV pentameric complex (gH/gL/pUL128/pUL130/pUL131)	Double-blind, randomized, placebo-controlled, dose escalation multicentre study conducted November 2013–March 2017 (NCT01986010) Adler S.P. et al. [75]	V160 had acceptable safety profile. Neutralising antibody levels and T-cell responses in seronegative subjects resembled those following natural infection.
CMV subunit gB glycoprotein/MF59 adjuvant	Double-blind, randomized, placebo-controlled, Phase II trial of safety and efficacy conducted July 2006–June 2013 (NCT00133497) Bernstein D.I. et al. [76]	Vaccine was safe and immunogenic. Efficacy compared to placebo after three doses estimated at 42.9%.
Two component alphavirus replicon vaccine expressing CMV proteins gB, pp65, and IE1 (AVX601)	Double-blind, randomized, placebo-controlled, Phase 1 trial of safety and immunogenicity conducted March 2007–June 2007 (NCT00439803) Bernstein D.I. et al. [77]	Vaccine was safe with mild to moderate local reactogenicity which was short-lived following IM injection. Neutralizing antibody and multifunctional T cell responses induced.
Live Towne/Toledo chimera vaccines	Phase 1 dose-escalation study without a placebo conducted October 2011–October 2014 (NCT01195571) Adler S.P. et al. [78]	No serious local or systemic reactions. Immunogenicity varied depending upon chimera but was generally low.
Attenuated poxvirus modified vaccinia Ankara (MVA) expressing pp65, IE1-exon4, and IE2-exon5 Triplex vaccine	Open label, single-arm, dose-escalating Phase 1 clinical trial La Rosa et al. [79]	Vaccine was well tolerated in healthy adults and was highly immunogenic.
Bivalent CMV DNA vaccine VCL-CB01 comprising two plasmids encoding pp65 and gB formulated with poloxamer CRL1005 and benzalkonium chloride	Phase 1, multicentre, open-label, dose-escalating trial Wloch et al. [80]	No serious adverse advents but low-grade adverse events were common. Immunogenicity documented in 45.5% of CMV-seronegative subjects and in 25.0% of CMV-seropositive subjects.
